# Visualizing epithelial expression of EGFR in vivo with distal scanning side-viewing confocal endomicroscope

**DOI:** 10.1038/srep37315

**Published:** 2016-11-22

**Authors:** Xiyu Duan, Haijun Li, Juan Zhou, Quan Zhou, Kenn R. Oldham, Thomas D. Wang

**Affiliations:** 1 Department of Biomedical Engineering, University of Michigan, Ann Arbor, MI, 48109, USA; 2 Department of Internal Medicine, University of Michigan, Ann Arbor, MI, 48109, USA; 3 Department of Mechanical Engineering, University of Michigan, Ann Arbor, MI, 48109, USA

## Abstract

Confocal endomicroscopy is an emerging imaging technology that has recently been introduced into the clinic to instantaneously collect “optical biopsies” *in vivo* with histology-like quality. Here, we demonstrate a fast scanner located in the distal end of a side-viewing instrument using a compact lens assembly with numerical aperture of 0.5 to achieve a working distance of 100 μm and field-of-view of 300 × 400 μm^2^. The microelectromechanical systems (MEMS) mirror was designed based on the principle of parametric resonance and images at 5 frames per second. The instrument has a 4.2 mm outer diameter and 3 cm rigid length, and can pass through the biopsy channel of a medical endoscope. We achieved real time optical sections of NIR fluorescence with 0.87 μm lateral resolution, and were able to visualize *in vivo* binding of a Cy5.5-labeled peptide specific for EGFR to the cell surface of pre-cancerous colonocytes within the epithelium of dysplastic crypts in mouse colon. By performing targeted imaging with endomicroscopy, we can visualize molecular expression patterns *in vivo* that provide a biological basis for disease detection.

Many human diseases originate within the epithelium of hollow organs and ducts^1^. This thin layer of tissue has dimensions of only a few hundred microns^2^, a simple repetitive architecture^3^, and intense self-renewal kinetics^4^. Optical sectioning is being developed to visualize molecular expression patterns unique to biological processes that drive disease progression with sub-cellular resolution. For *in vivo* imaging, these instruments must be made sufficiently small in size and fast in speed. Confocal endomicroscopes have been developed for this purpose with front-viewing optics, and are now being used routinely in the clinic^5^,^6^. Front-view optics requires instrument placement perpendicular to the tissue surface. This can be difficult in areas where space to maneuver is limited, such as in colon, biliary tract, and pancreatic duct. Side-viewing optics may be more useful for these imaging applications.

Confocal endomicroscopy is an emerging imaging technology that is being developed to instantaneously collect “optical biopsies” *in vivo* with histology-like quality^7^. These instruments use the core of a single mode optical fiber that acts as a “pinhole” to reject light scattered by tissue. A flexible fiber is used to deliver and collect light through a medical endoscope. Current clinical endomicroscopes are limited in imaging performance by simple scanning mechanisms that are either too slow in speed resulting in motion artifacts or too bulky in dimension requiring a proximal location that reduces resolution. A fast compact scanner placed in the distal end of the endomicroscope can provide improved performance and flexibility for instrument control. We have previously developed a miniature scanner to perform high speed *in vivo* imaging^8^. This design uses parametric resonance where drive signals are applied at frequencies near 2ω_o_/n (ω_o_ is the natural frequency of vibrational modes and n is an integer ≥1)^9^. These electrostatic devices were fabricated with compact dimensions using micro-electro-mechanical systems (MEMS) processes^10^.

In clinical confocal endomicroscopy, contrast has been generated using non-specific methods, such as reflectance^11^, endogenous fluorescence^12^, topically applied intra-vital dyes^13^, and intravenously injected fluorescein^14^. Targeted imaging agents have recently been developed that detect molecules that are specific to disease and have known biological function. These contrast agents require high resolution *in vivo* imaging to validate specific binding to the intended target. We have previously identified a peptide specific for domain 2 of EGFR^15^, a cell surface target that is overexpressed in up to 80% of human colonic adenomas^16^ and 97% of adenocarcinomas^17^. We have demonstrated targeted images from the mucosal surface of mouse colonic adenomas *in vivo* with topical administration using wide-field fluorescence endoscopy^15^. EGFR is also overexpressed in most epithelial-derived cancers, including breast^18^, esophagus^19^, head and neck^20^, lung^21^, and pancreas^22^, and is the target for a number of antibody and small molecule therapies, such as cetuximab and gefitinib^23^,^24^. Here, we aim to systemically administer a peptide specific for EGFR, and demonstrate use of a high resolution side-viewing endomicroscope to validate specific cell surface binding to dysplastic colonocytes *in vivo*.

## Results

### Side-viewing confocal endomicroscope

**Figure 1:**
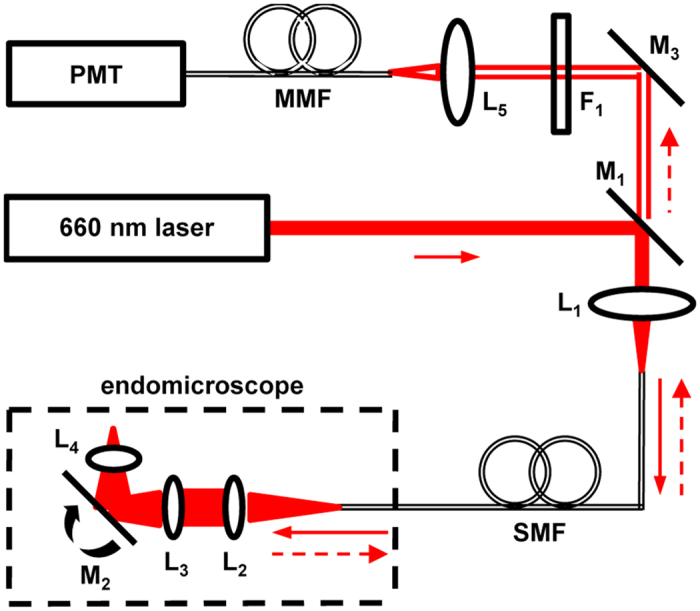
Schematic. Optical configuration of side-viewing endomicroscope. Details provided in text. Key: PMT – photomultiplier tube, MMF – multi-mode fiber, SMF – single mode fiber, L – lens, F – filter, M – mirror.

**Figure 2:**
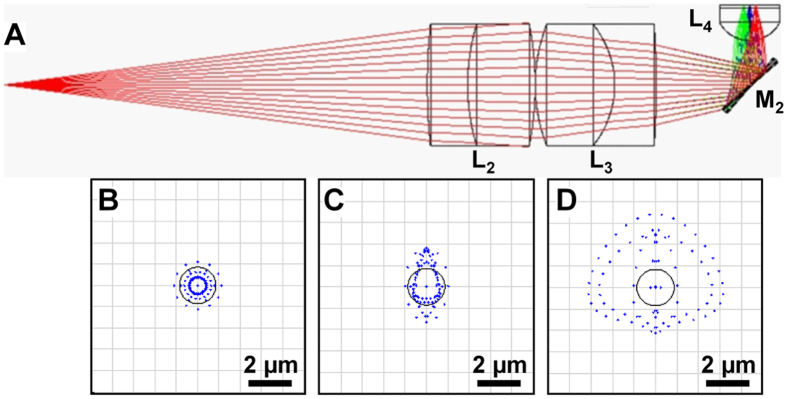
Design of optics. (**A**) A ray-trace simulation was used to design the distal optics (L_2_-L_4_). (**B**–**D**) Spot size with RMS radius of 0.632, 0.794 and 1.94 μm can be achieved at a distance of 0, 75 and 150 μm away from the center of the FOV. The radius of the Airy disk is 0.59 μm.

The schematic for a side-viewing endomicroscope is shown, Fig. 1A. A laser provides excitation at λ_ex_ = 660 nm. We performed ray trace simulations to design the optics to achieve diffraction-limited resolution with a working distance of 100 μm, Fig. 2A–D. This imaging depth is representative of the thickness of the epithelium in mouse colon. Only lenses with outer diameter <3 mm were considered for use. Commercially available lenses were chosen to demonstrate proof-of-concept. The illumination beam is reflected by a dichroic mirror M_1_, focused by lens L_1_ into a 2 meter long single mode optical fiber (SMF), collimated by L_2_, and focused by L_3_. The beam is reflected 90° for side-viewing by the MEMS mirror M_2_ into a solid immersion lens L_4_ that provides contact with tissue and index matching. Near-infrared (NIR) fluorescence is collected by the same optics, transmits through the SMF optical fiber, passes through the dichroic M_1_, reflects off the static mirror M_3_, passes through band pass filter F_1_ (696–736 nm), focused by lens L_5_ into a 1 meter long multimode fiber (MMF), and transmits to the photomultiplier tube (PMT) detector. This design produced an effective numerical aperture (NA) of 0.5 with a field-of-view (FOV) of 300 × 400 μm^2^. Full instrument details are provided in Methods.

**Figure 3:**
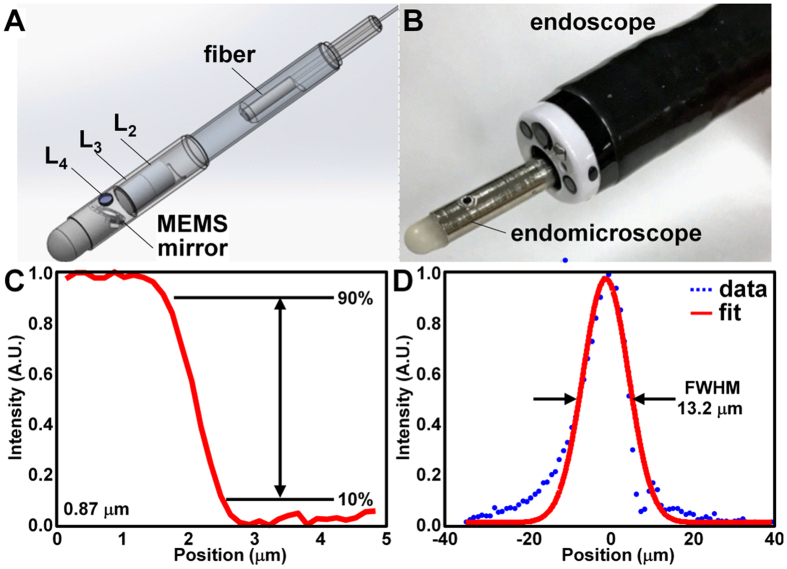
Side-viewing endomicroscope. (**A**) CAD drawing shows how instrument is packaged. (**B**) Dimensions allow for endomicroscope to pass through a 6 mm diameter biopsy channel of Olympus GIF XTQ160 endoscope. (**C**) Lateral resolution of 0.87 μm was measured from width of transition from 10% to 90% of maximum intensity from profile across knife-edge target. (**D**) Axial resolution of 13.2 μm was measured from full-width-at-half-maximum (FWHM) of intensity profile produced by translating a plane mirror in axial direction at a neutral position of 100 μm.

The packaging scheme for the mirror, optics, and fiber is shown, Fig. 3A. The assembled endomicroscope is shown passed through a 6 mm diameter biopsy channel of a medical endoscope, Fig. 3B. The rigid end of the endomicroscope was soaked in distilled water overnight to ensure an adequate seal. No leaks were observed after more than 20 *in vivo* uses in mouse colon.

**Figure 4:**
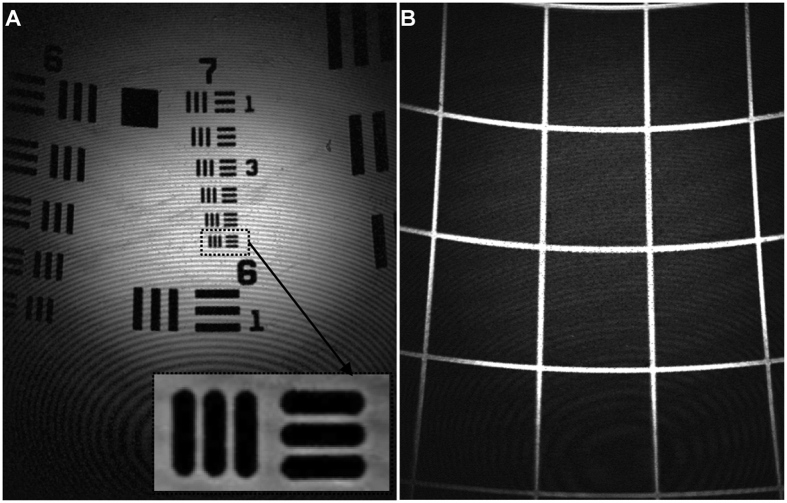
Image parameters. (**A**) Reflectance image of standard resolution target (USAF 1951) qualitatively supports lateral resolution of <2 μm. Vertical and horizontal bars from group 7, element 6 are clearly seen in expanded view of box. (**B**) Reflectance image of grid with 100 × 100 μm^2^ squares shows a field-of-view of ∼300 × 400 μm^2^. Note: interference patterns seen in either image result from multiple reflections between the outer face of the distal lens L_4_ and the reflective surface of the resolution target. This condition does not occur when imaging tissue. The corners of the image appear darker due to curvature of the image plane when scanning. The focal spots in the corners are offset by 7 μm compared to the center. Images were collected at a working distance of ~100 μm.

We plot the intensity profile along a knife-edge target to measure a lateral resolution of 0.87 μm, defined as the distance spanned by 10% to 90% of maximum intensity, Fig. 3C. We measured the signal collected while vertically translating a reflective surface, and measured a full-width-at-half-maximum (FWHM) of 13.2 μm for the axial resolution, Fig. 3D. We collected reflectance images from a standard resolution target to qualitatively estimate a lateral resolution <2 μm (group 7, element 6 in expanded view of box), Fig. 4A. A reflectance image from a target with 100 × 100 μm^2^ square grids showed a FOV of 300 × 400 μm^2^, Fig. 4B. Mild distortion in the image results from use of the scan mirror that rotates about separate axes, and can be corrected with a look-up table, if needed.

### MEMS scanner

**Figure 5:**
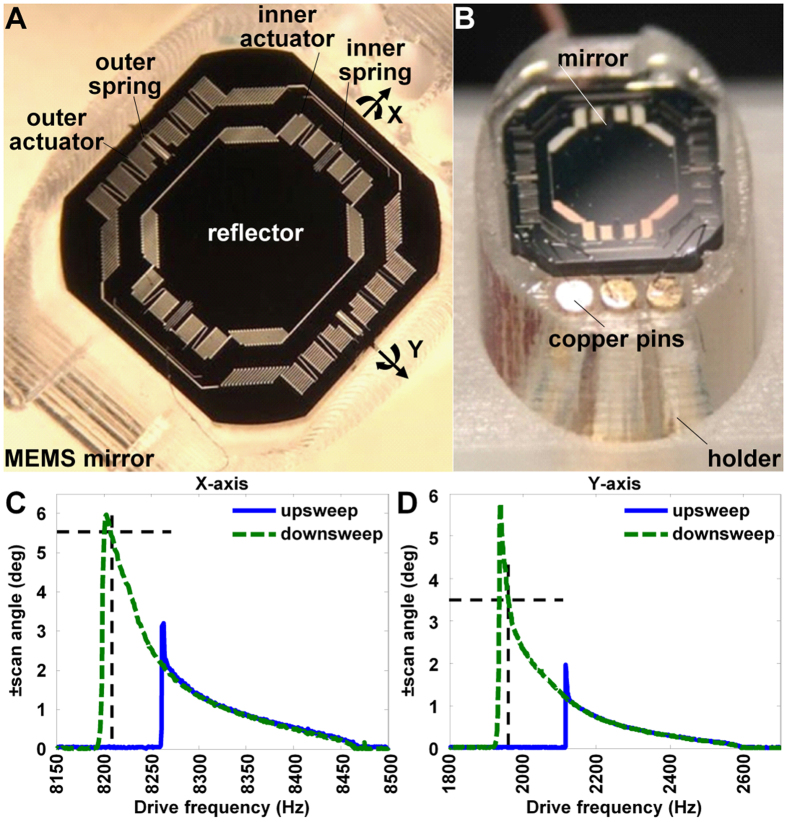
MEMS scanner. (**A**) An aluminum-coated reflector is mounted on a gimbal frame and is driven by orthogonal sets of electrostatic comb-drive actuators coupled to inner and outer torsional springs to produce rapid Lissajous scanning. (**B**) Mirror is secured by custom holder using copper pins as contacts to wire bond electrical connections. Frequency response shows mechanical scan angles for (**C**) inner and (**D**) outer axes with a drive voltage of 60 V_pp_ with either an upsweep (low-to-high) or downsweep (high-to-low) in frequency. The inner and outer axes were driven at 8210 Hz and 1960 Hz to achieve mechanical deflections of 5.5 and 3.5 degrees, respectively.

We have previously designed, fabricated, and verified a compact 2D MEMS scanner with chip size of 3 × 3 mm^2^ and a 1.8 mm diameter circular reflector, Fig. 5A^25^. We reduced the diagonal dimension to 3.5 mm by cutting the four corners to match the reduced dimensions of the side-viewing endomicroscope. The reflector is mounted on a gimbal frame to minimize cross-talk between axes. Orthogonal sets of electrostatic comb-drive actuators are coupled to inner and outer torsional springs designed to achieve resonant frequencies near ~4 and ~1 kHz, respectively. A Lissajous scan pattern is created with a frame rate ≥5 Hz to cover 400 × 400 pixels. Aluminum was sputtered on the front-side silicon surface to achieve reflectivity >90% between 400 and 700 nm. We mounted the mirror onto a MEMS holder using copper pins to wire bond the electrical contacts, Fig. 5B. We measured the angular deflection of the MEMS scanner in either axis to characterize the frequency response to a sine-wave drive signal at 60 V_pp_. For *in vivo* imaging, we used frequencies of 8210 and 1960 Hz to produce tilting frequencies at 4105 Hz and 985 Hz in the X and Y axes, respectively, Fig. 5C,D. We packaged the assembly in a 4.2 mm diameter stainless steel tube. The distance between lens L_3_ and the 2D resonant mirror M_2_ was adjusted to achieve a working distance of 100 μm. For *in vivo* imaging, the endomicroscope was mounted onto a motorized rotational and translational platform to control the position of the distal end.

### *In vivo* imaging of EGFR expression in mouse colonic adenoma

**Figure 6:**
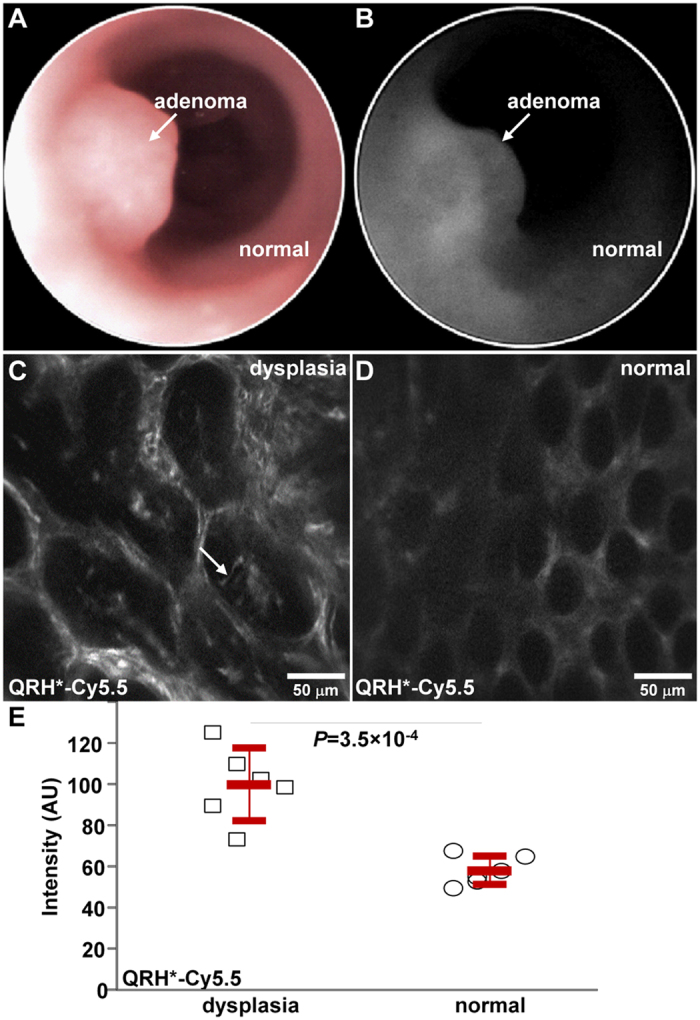
Imaging of mouse colonic dysplasia. (**A**) White light image of *CPC;Apc* mouse colon collected *in vivo* with wide-field endoscope shows location of spontaneous adenoma (arrow). (**B**) Maximum uptake of the NIR-labeled EGFR peptide QRH*-Cy5.5 is seen from the adenoma ~90 minutes after systemic administration. From the *in vivo* confocal images, (**C**) increased fluorescence intensity is seen from cell surface of dysplastic colonocytes (arrow) while (**D**) minimal staining is seen in normal crypts. Real time video (visualization) of side-viewing endomicroscope moving from adenoma to normal is shown. (**E**) A significantly greater mean fluorescence intensity was measured from dysplasia compared with that of adjacent normal, 107 ± 19 versus 62 ± 8 AU, from a group of n = 6 mice, *P* = 3.5 × 10^−4^ by paired two-way t-test.

We performed *in vivo* imaging first with wide-field endoscopy using white light illumination in the colon of n = 6 *CPC;Apc* mice, Fig. 6A. These genetically-engineered animals develop adenomas spontaneously on the side wall of the colon that can be difficult to access with front-viewing optics^26^. We defined the distance between the endoscope tip and the anus and the clockwise location of the adenoma as landmarks to guide subsequent placement of the endomicroscope. We performed an intraperitoneal injection of Cy5.5-labeled peptide QRH* at a concentration of 300 μM in a volume of 600 μL and molecular weight = 1336.48. After ∼90 minutes, we observed peak fluorescence intensity from the adenoma, Fig. 6B. We then inserted the side-viewing endomicroscope, and used a motorized rotational and translational platform to position the distal tip onto the adenoma using the landmarks as a guide, Fig. S1. Optical sections were collected *in vivo* using a laser power of 1.5 mW. We observed strong binding of the EGFR peptide to the cell surface of dysplastic colonocytes (arrow), Fig. 6C, and minimal staining to normal colonic epithelium, Fig. 6D. A video (visualization) shows real time images being collected at 5 frames per sec while the side-viewing endomicroscope moves from an adenoma to normal colonic mucosa. We quantified each image by measuring the average fluorescence intensity from n = 5 regions of interest (ROI) with dimensions of 20 × 20 pixels that represented a range of intensities for dysplasia and adjacent normal. We found significantly greater mean fluorescence intensities *in vivo* for dysplasia compared with normal colonic mucosa, Fig. 6E.

### Validation of EGFR expression in mouse colonic epithelium

**Figure 7:**
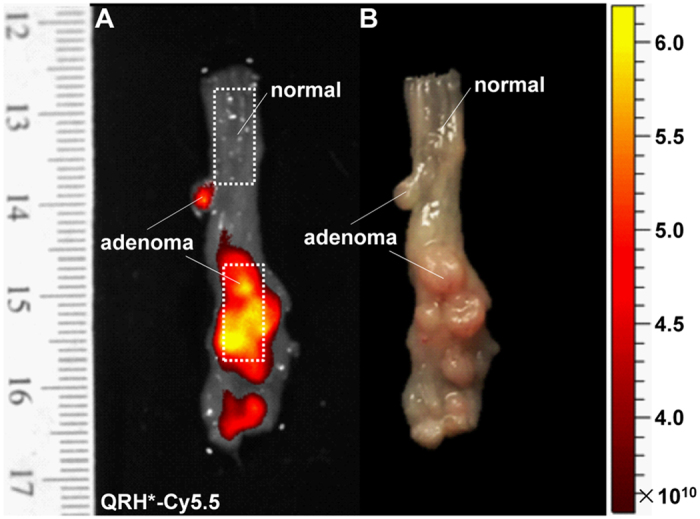
Uptake of EGFR peptide in adenomas. (**A**) Representative NIR fluorescence image collected *ex vivo* show selective uptake of QRH*-Cy5.5 in adenomas compared with adjacent normal colonic mucosa. Intensities were measured from the ROIs defined by the dotted rectangles. We calculated a mean T/B ratio of 3.47 ± 1.87 from the group of n = 6 mice. (**B**) Co-registered white light image of exposed mucosal surface confirms locations of adenomas.

After completion of endomicroscopy, we euthanized the animals and performed macroscopic imaging of excised colonic mucosa to validate selective uptake of the EGFR peptide in adenomas. Representative NIR fluorescence images were collected using a Cy5.5 filter set (λ_ex_ = 675 nm excitation, λ_em_ = 720 nm emission, 0.05 sec exposure), and showed increased fluorescence intensities from adenomas compared with adjacent normal colonic mucosa, Fig. 7A. A ruler was placed next to the specimen to confirm the distance of the adenomas from the anus to register the images. Fluorescence intensities were measured from ROI (dotted boxes) around dysplasia (target) and adjacent normal (background). The target-to-background (T/B) ratio for each mouse was calculated by dividing the average intensity from a ROI of dysplasia with that from normal. We measured a T/B ratio of 3.47 ± 1.87 from n = 6 mice. Co-registration with white light images further confirmed the location of the adenomas, Fig. 7B.

**Figure 8:**
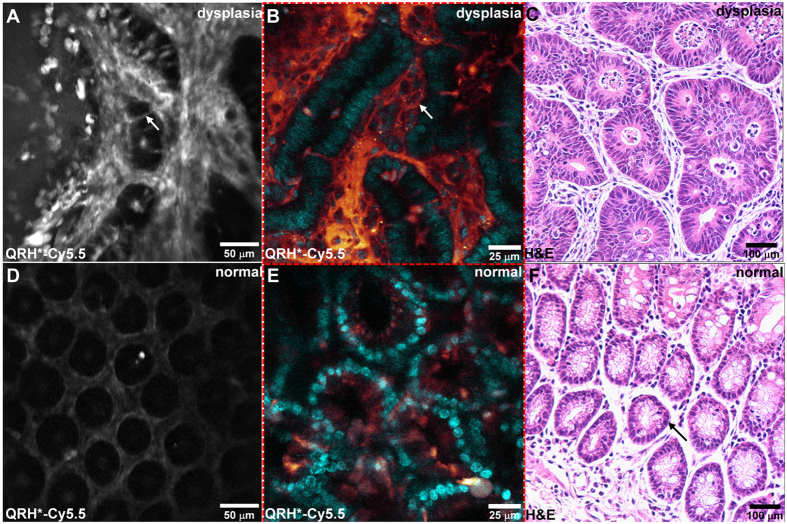
Immunofluorescence of mouse colon *ex vivo*. Strong fluorescence intensity is seen from binding of the EGFR peptide QRH*-Cy5.5 to the cell surface (arrow) of dysplastic colonocytes using the (**A**) side-viewing confocal endomicroscope and (**B**) a conventional tabletop confocal microscope. (**C**) Representative histology (H&E) for dysplasia. Minimal fluorescence intensity is seen for normal colonocytes using the (**D**) side-viewing confocal endomicroscope and (**E**) a conventional tabletop confocal microscope. (**F**) Representative histology (H&E) for normal colonic mucosa.

We then validated specific binding of the EGFR peptide to pre-cancerous crypts using our endomicroscope on mouse colonic mucosa *ex vivo* and compared our images with that collected using a tabletop confocal microscope. Increased intensity (arrow) is seen at the cell surface of dysplastic colonocytes, Fig. 8A,B. We observed greatly reduced signal from the EGFR peptide for normal colon, Fig. 8D,E. Representative histology (H&E) is shown for dysplasia and normal, Fig. 8C,F.

#### Panorama view

We rapidly created mosaic images of colonic mucosa to enlarge the FOV. We used the motorized platform to rapidly rotate and translate the endomicroscope along the colon lumen. ~1 hour after injection of Cy5.5, we collected a panorama image of normal colonic mucosa *in vivo* while translating the endomicroscope a distance of 1 mm and rotating the instrument by 90°, Fig. S2A. We simulated the *in vivo* anatomy of colon by warping the image in custom software, Fig. S2B.

## Discussion

Here, we demonstrate a side-viewing confocal endomicroscope that uses a fast, compact, distal scanner to collect real time *in vivo* optical sections in colonic epithelium. Using MEMS technology, we were able to scale down the dimensions of the device for efficient packaging and perform rapid beam scanning. This capability provides significantly improved imaging performance in terms of speed and resolution compared with other endomicroscope designs currently used in the clinic. The instrument showed stable operation at 5 frames per second with minimal interference from mechanical vibrations while imaging. With further optimization, we can achieve frame rates as high as 10 per second. Using a Cy5.5 labeled peptide, we were able to collect bright optical sections of EGFR expression in mouse colonic epithelium at a depth of ∼100 μm. After completion of packaging, the scanner performed reliably, and was able to collect images continuously without any noticeable phase shifts at fixed drive frequencies. We fine tuned the phase at the beginning of each experiment. Position sensing of the MEMS device may be needed for use in more versatile environments.

We collected NIR fluorescence images with sub-micron lateral resolution. At an imaging depth of 100 μm, we were able to visualize binding of a Cy5.5-labeled peptide specific for EGFR to the cell surface of colonocytes within the epithelium of dysplastic crypts. The extra-cellular domain of EGFR in human and mouse has 97.5% homology, and is 100% conserved in domain 2 where the EGFR peptide binds^27^. We used the *CPC;Apc* mouse that has been genetically engineered to sporadically delete an *APC* gene^26^, which is mutated in >80% of human colorectal carcinomas^28^. Adenomas protrude spontaneously from the side wall in the distal colon where they are more readily accessible to imaging with a side-viewing rather than front-viewing endomicroscope. EGFR is overexpressed in the vast majority of human colonic adenomas^16^, thus may serve as an early target for detection of colorectal cancer. These results were validated *ex vivo* on macroscopic imaging and with immunofluorescence using a conventional tabletop confocal microscope with the EGFR peptide and a known EGFR antibody.

We used a peptide to visualize EGFR expression in colonic adenomas because of their small size and low molecular weight by comparison to antibodies. These properties result in better vascular permeability, enhanced tissue diffusion, and greater tumor penetration for improved targeting^29^. Also, peptides clear rapidly for reduced background and less biodistribution in non-target tissues^30^. Peptides have less potential for immunogenicity than antibodies, which allows for repetitive use^31^. We used Cy5.5, a NIR fluorophore, to label the peptide to maximize imaging depth, avoid hemoglobin absorption, reduce tissue scattering, and minimize autofluorescence background^32^. By performing endomicroscopy with targeted imaging agents, we can visualize molecular expression patterns *in vivo* that provide a biological basis for disease detection. By comparison with static biopsies from *ex vivo* specimens, real time *in vivo* imaging may provide a more robust approach to evaluate pathology of neoplastic lesions and has potential for use in point-of-care diagnostics. This approach may also identify patients most likely to respond to targeted therapies.

By comparison, the Optiscan ISC-1000^®^ uses a tuning fork mechanism to mechanically scan a single mode optical fiber. *In vivo* images are acquired at a slow frame rate of 0.8 to 1.6 per second, and imaging can be limited by motion artifacts from gut peristalsis, respiratory motion, and heart beats^33^. The Mauna Kea Technologies Cellvizio^®^ uses a flexible optical fiber bundle that ranges in diameter from 0.3 to 4.2 mm. The smaller probes can be used to image small caliber hollow organs, such as the biliary tract and pancreatic duct^34^. Lateral scanning is performed using galvo mirrors located at the proximal end. The image resolution is limited by the number of individual fibers in the bundle^33^. The fibers are hexagonally packed, and the space in between fibers constitutes a large fraction of area where no light transmits. The fiber cores are multi-mode, and not optimal for rejecting scattered light. Side-viewing endomicroscopy has also been demonstrated using a 50 mm long graded-index (GRIN) lens coupled to a prism that deflects the beam at 90° 35. Scanning is also performed at the proximal end using a bulky galvo mirror. Long rigid GRIN lenses incur chromatic aberrations that may need correction when imaging fluorescence, and are not compatible with flexible medical endoscopes. Side-viewing has also been used in a spectrally encoded confocal endomicroscope (SECM)^36^. This design uses a diffraction grating to spatially deflect different wavelengths of light delivered from a broad spectrum illumination source. Images are formed by mechanically rotating and translating the optics. This technique is limited to collection of reflectance, and has not been used to image molecular expression.

To our knowledge, we have demonstrated the first side-viewing confocal endomicroscope using a MEMS-based scanner. These devices can provide fast imaging speeds in a small footprint. Scanning in the distal tip of the endomicroscope provides greater flexibility for image formation. Other MEMS-based instruments include a 7 mm diameter front-viewing confocal endomicroscope that uses an electrothermal mirror. Lateral scanning is performed with ±26° of deflection, and a FOV of 180 × 180 mm^2^ is produced^37^. A separate electrothermal actuator consisting of 4 bimorphs provides ~400 μm of out-of-plance displacement. The lateral and axial resolutions were found to be 1 and 7 μm, respectively. Also, a 5.5 mm diameter front-viewing dual axes confocal endomicroscope was developed using a lateral scanning electrostatic MEMS mirror^38^. The instrument achieved a FOV of 362 × 212 μm^2^, and a micromotor was used to image at a depth of 140 μm. The lateral and axial resolution was 5 and 6.5 μm, respectively, and images were collected at 5 frames per sec. A handheld 12 mm diameter dual axes confocal microscope has been demonstrated using a lateral scanning electrostatic MEMS mirror with a resonance frequency of ~200 Hz^39^. Line scanning was performed to improve the imaging speed to 16 frames per sec. A custom, multi-element objective lens was to achieve a lateral and axial resolution of 1.1 and 2 μm, respectively, with a FOV of 300 × 300 μm^2^.

Our endomicroscope design has potential to be used as an adjunct to wide-field endoscopy by providing instantaneous “optical” biopsy. Targeted imaging can be used to stage early cancer, guide choice for therapy, and monitor therapeutic response. Furthermore, this instrument can quickly evaluate numerous tissue sites to minimize the number of physical biopsies collected, risk for bleeding, and procedure time. It may also be useful for detecting pre-malignant lesions that may otherwise be missed because of flat appearance, focal size, and patchy distribution. In the future, we can add the capability to perform depth scanning by using a MEMS device that performs out-of-plane displacement^8^. We found side-viewing to be effective for *in situ* imaging of adenomas located on the side wall of mouse colon that would otherwise be inaccessible with a front-viewing geometry. For the same reason, this instrument can potentially be useful in the clinic to provide real-time pathology and assess important clinical conditions, such as flat colonic adenomas, indeterminant biliary strictures, and pancreatic intraepithelial (PanIN) lesions.

## Methods

### Side-viewing confocal endomicroscope

We performed ray-trace simulations (ZEMAX, ver 13) to design optics for the side-viewing endomicroscope. Our design criteria included diffraction-limited resolution on axis with working distance of 100 μm. We used a solid-state diode laser (660-S, Toptica Photonics) to deliver fluorescence excitation at λ_ex_ = 660 nm. The beam is reflected by a dichroic mirror M_1_ (FF685-Di02–25 × 36, Semrock) and focused by lens L_1_ (f = 11 mm, C220TMD-B, Thorlabs) into a 2 meter long single mode optical fiber (SMF, SM600, Thorlabs). The illumination is collimated by lens L_2_ (f = 12 mm, 45–262, Edmund Optics) to a beam diameter of 3 mm and focused by lens L_3_ (f = 6 mm, 45–089, Edmund Optics). The focused beam is reflected 90° by the 2D resonant MEMS mirror M_2_ into a solid immersion lens L_4_ with (f = 1 mm, OD 1.5 mm, 65–261, Edmund Optics). For L_2_, L_3_, and L_4_, only commercially available lenses with outer diameter <3 mm were considered for endoscope compatibility.

NIR fluorescence (dashed red arrows, Fig. 1) is collected by the same optics and travels in the opposite direction along the same path, is descanned by M_2_, and focused into the same single mode fiber (SMF). After exiting the fiber, fluorescence is collimated by L_1_, passes through the dichroic M_1_, reflected by M_3_, and passes through a band pass filter F_1_ (FF01-716/40-25, Semrock) that transmits from 696 to 736 nm with >93% transmission efficiency, and is focused by lens L_5_ (f = 6.24 mm, C110TMD-B, Thorlabs) into a 1 meter long multimode fiber (MMF, FT400UMT, Thorlabs). The multimode fiber transmits fluorescence to a photomultiplier tube (PMT) detector (H7422–40, Hamamatsu). The PMT signal is amplified by a high-speed current amplifier (59–178, Edmund Optics). A high-speed multi-function data acquisition board (National Instruments, PCI-6115) is used to digitize the amplified PMT signal. The same board was used to generate control signals to drive the 2D MEMS scanner. The data acquisition board was controlled using custom software (LabVIEW, National Instruments).

### MEMS scanner

A compact scanner was designed and fabricated using micro-electro-mechanical systems (MEMS) processes. The reflector has diagonal dimensions of 3.5 mm, and is mounted on a gimbal frame to minimize vibrational cross-talk between orthogonal axes. The mirror is rotated about either axis by drive signals applied to panels of electrostatic comb-drive actuators coupled to inner and outer torsional springs. Custom software was developed in LabView to drive the MEMS scanner and reconstruct the image by remapping the time series signal in a 2D matrix.

At the center of the FOV of the endomicroscope, we measured a lateral resolution of ∼0.90 μm. To satisfy the Nyquist sampling criteria, we must sample with spacing <0.45 μm between pixels. We used different scanning parameters for *in vivo* versus *ex vivo* images. For *in vivo* imaging, we used tilting frequencies of 4105 and 985 Hz to create a Lissajous scan pattern with 400 × 400 pixels at 5 frames per sec to cover a FOV of 300 × 300 μm^2^. The pixel spacing is 0.75 μm, and we are under sampling a little to maintain a higher frame rate to incur less motion artifact. For *ex vivo* imaging, we used frequencies of 4105 and 976 Hz to create a scan pattern with 1000 × 1000 pixels at 1 frame per sec to cover a FOV of 300 × 400 μm^2^. The pixel spacing is ≤0.4 μm, and meets the Nyquist sampling criteria.

We used computer-aided design (CAD) software (Solidworks, ver 2015) to design the housing to package the optics and scanner. We used an stainless steel tube with an outer diameter (OD) of 3.4 mm and an inner diameter (ID) of 3 mm to center and align the lenses L_2_ and L_3_, and a second stainless steel tube with OD 4.2 mm and ID 3.4 mm to enclose the MEMS scanner and lens L_4_. We fabricated the MEMS holder with OD 3.8 mm using M3 Crystal, an acrylic based resin with high strength and stiffness. We used a high precision 3D printer (ProJet 3500HD MAX, 3DSystems) with 16 μm resolution. A total of 3 copper pins were inserted for wire bonding. The distance between L_3_ and M_2_ was adjusted to achieve a working distance of 100 μm. The position of M_2_ was adjusted using a 6-axis precision manipulator to ensure that the reflected beam was at normal incidence to L_4_. After assembly, the probe was sealed using epoxy (G14250, Thorlabs).

We verified the dimension of the packaged endomicroscope by passing it through the 6 mm diameter instrument channel of a medical endoscope (Olympus GIF XTQ160). Prior to use, the distal tip of the endomicroscope was soaked in distilled water overnight to ensure an adequate seal. For *in vivo* imaging, the endomicroscope was mounted onto a rotational and translational platform driven by a motorized linear stage (CONEX-LTA-CC, Newport) and a stepper motor (324, Adafruit) to precisely control the distal end of the endomicroscope in the mouse colon.

### *In vivo* imaging of EGFR expression in mouse colonic adenoma

All experimental procedures were performed in accordance with relevant guidelines and regulations of the University of Michigan, and the mouse imaging studies and all experimental procedures were conducted with approval by the University Committee on the Use and Care of Animals (UCUCA). The mice were housed in pathogen-free conditions and supplied water ad libitum under controlled conditions of humidity (50 ± 10%), light (12/12 hour light/dark cycle) and temperature (25 °C). We have previously developed a peptide that is specific for domain 2 of EGFR^15^. We synthesized the monomeric linear peptide with sequence QRHKPRE, hereafter QRH*, with linker GGGSK using standard Fmoc-mediated solid-phase synthesis^40^. QRH* was labeled with a water soluble NIR fluorophore using sulfo-Cy5.5- N-hydroxysuccinimide ester (Lumiprobe LLC). After lyophilization, the peptides were stored at −80 °C.

We used a *CPC;Apc* mouse that spontaneously develops adenomas in the distal colon where they are accessible by the side-viewing endomicroscope. During imaging, 12-week-old *CPC;Apc* mice were anesthetized with 2% isoflurane (Fluriso; MWI Veterinary Supply Co.). We first used a wide-field small animal endoscope (27030BA, Karl Storz Veterinary Endoscopy) with white light illumination to examine the colon for presence of grossly visible adenomas. We waited ∼90 minutes after intraperitoneal injection of QRH*-Cy5.5 to achieve maximum target uptake. The distal tip of the endomicroscope was placed into the colon of anesthetized mice. The insertion depth and angle were controlled using the translational stage and the rotational fixture in order to image normal colonic mucosa and adenomas at the same locations as first detected on the wide-field endoscope.

Live video streams were collected ~100 μm below the mucosa surface at 5 frames/sec using a laser power of 1.5 mW. Individual frames with minimal motion artifacts were identified. After completion of imaging, the mice were euthanized, and the colon was harvested for *ex vivo* imaging. The optical window of probe was positioned directly en face over the exposed mucosal surface of resected colon to optimize contact. Images were collected with the same laser power as that used *in vivo*. Previously identified landmarks were used to confirm the locations of the adenomas. The colon was fixed in 10% buffered formalin and paraffin embedded for routine histology (H&E). Images and tissues were collected and analyzed in a total of n = 6 mice. One representative image was selected from either dysplasia or normal for each mouse. We quantified each image by measuring the average fluorescence intensity from n = 5 regions of interest (ROI) with dimensions of 20 × 20 pixels that represented a range of intensities for dysplasia and adjacent normal.

### Validation of EGFR expression in mouse colonic epithelium

After completion of imaging, the animals were euthanized, and the colon was excised, flushed with PBS, and divided longitudinally for macroscopic imaging (IVIS 200, Caliper Life Sciences), to validate specific uptake by the EGFR peptide. NIR fluorescence images were collected using a Cy5.5 filter with λ_ex_ = 675 nm excitation and 720 nm emission with 0.05 sec exposure. A ruler was placed next to the specimen to determine the distance from the anus for registration with the endoscopy and histology images. Whole mount tissue was imaged with a conventional tabletop confocal microscope (Leica TCS SP5 Microsystems) using Cy5.5 and DAPI filters. The colon was then fixed in 10% buffered formalin and processed for routine histology (H&E).

### Panorama view

The side-viewing endomicroscope can be rapidly rotated and translated in the lumen of the colon to enlarge the dimensions of the images collected using the motorized platform. We first injected Cy5.5, and waited approximately 1 hour for the dye to circulate. We collected images in a video stream of normal colonic mucosa *in vivo* while translating the endomicroscope a distance of 1 mm and rotating the instrument by 90°. We stitched together individual images to form a mosaic and warped the composite image to simulated the *in vivo* anatomy of colon using custom software (Matlab, Mathworks Inc).

## Additional Information

**How to cite this article**: Duan, X. *et al.* Visualizing epithelial expression of EGFR in vivo with a distal scanning side-viewing confocal endomicroscope. *Sci. Rep.* **6**, 37315; doi: 10.1038/srep37315 (2016).

**Publisher’s note:** Springer Nature remains neutral with regard to jurisdictional claims in published maps and institutional affiliations.

## Supplementary Material

Supplementary Information

Supplementary Video

## References

[b1] Torre L. A., Bray F., Siegel R. L., Ferlay J., Lortet-Tieulent J. *et al.* Global cancer statistics, 2012. CA Cancer J Clin. 65, 87–108 (2015). 25651787 10.3322/caac.21262

[b2] Wang T. D., Van Dam J., Crawford J. M., Preisinger E. A., Wang Y. *et al.* Fluorescence endoscopic imaging of human colonic adenomas. Gastroenterology. 111, 1182–1191 (1996). 8898631 10.1053/gast.1996.v111.pm8898631

[b3] Clevers H. The intestinal crypt, a prototype stem cell compartment. Cell. 154, 274–284 (2013). 23870119 10.1016/j.cell.2013.07.004

[b4] Barker N., Ridgway R. A., van Es J. H., van de Wetering M., Begthel H. *et al.* Crypt stem cells as the cells-of-origin of intestinal cancer. Nature. 457, 608–611 (2009). 19092804 10.1038/nature07602

[b5] Kiesslich R., Burg J., Vieth M., Gnaendiger J., Enders M. *et al.* Confocal laser endoscopy for diagnosing intraepithelial neoplasias and colorectal cancer *in vivo*. Gastroenterology. 127, 706–713 (2004). 15362025 10.1053/j.gastro.2004.06.050

[b6] Wang T. D., Friedland S., Sahbaie P., Soetikno R., Hsiung P. L. *et al.* Functional imaging of colonic mucosa with a fibered confocal microscope for real-time *in vivo* pathology. Clin Gastroenterol Hepatol. 5, 1300–1305 (2007). 17936692 10.1016/j.cgh.2007.07.013 PMC2104519

[b7] Wang T. D. Confocal Microendoscopy – From the Bench to the Bedside. Gastrointestinal Endoscopy. 62, 696–697 (2005). 16246681 10.1016/j.gie.2005.06.002 PMC2169357

[b8] Li H., Qiu Z., Duan X., Oldham K. R., Kurabayashi K. *et al.* A Monolithically-Integrated 3D MEMS Scanner for Switchable Real-time Vertical/Horizontal Cross-sectional Imaging in Dual Axes Confocal Endomicroscope. Optics Express. 24, 2145–2155 (2016). 26906790 10.1364/OE.24.002145 PMC5802237

[b9] Turner K. L., Miller S. A., Hartwell P. G., MacDonald N. C., Strogatz S. H. *et al.* Five parametric resonances in a microelectromechanical system. Nature. 396, 149–152 (1998).

[b10] Lee K., Krisnamoorthy K., Yu K. & Solgaard O. Single-crystalline silicon micromirrors actuated by self-aligned vertical electrostatic comb drives with piston-motion and rotational capabilities. Sensors and Actuators A: Physical. 114, 423–428 (2004).

[b11] Maitland K. C., Gillenwater A. M., Williams M. D., El-Naggar A. K., Descour M. R. *et al.* *In vivo* imaging of oral neoplasia using a miniaturized fiber optic confocal reflectance microscope. Oral Oncol. 44, 1059–1066 (2008). 18396445 10.1016/j.oraloncology.2008.02.002 PMC2673342

[b12] Thiberville L., Moreno-Swirc S., Vercauteren T., Peltier E., Cavé C. *et al.* *In vivo* imaging of the bronchial wall microstructure using fibered confocal fluorescence microscopy. Am J Respir Crit Care Med. 175, 22–31 (2007). 17023733 10.1164/rccm.200605-684OC

[b13] Thiberville L. & Salaün M. Bronchoscopic advances: on the way to the cells. Respiration. 79, 441–449 (2010). 20431326 10.1159/000313495

[b14] Wallace M. B., Meining A., Canto M. I., Fockens P., Miehlke S. *et al.* The safety of intravenous fluorescein for confocal laser endomicroscopy in the gastrointestinal tract. Aliment Pharmacol Ther. 31, 548–552 (2010). 20002025 10.1111/j.1365-2036.2009.04207.x

[b15] Zhou J., Joshi B. P., Duan X., Pant A., Qiu Z. *et al.* EGFR Overexpressed in Colonic Neoplasia Can be Detected on Wide-Field Endoscopic Imaging. Clin Transl Gastroenterol. 6, e101 (2015). 26181290 10.1038/ctg.2015.28 PMC4816258

[b16] Porebska I., Harlozińska A. & Bojarowski T. Expression of the tyrosine kinase activity growth factor receptors (EGFR, ERB B2, ERB B3) in colorectal adenocarcinomas and adenomas. Tumour Biol. 21, 105–115 (2000). 10686540 10.1159/000030116

[b17] Spano J. P., Lagorce C., Atlan D., Milano G., Domont J. *et al.* Impact of EGFR expression on colorectal cancer patient prognosis and survival. Ann Oncol. 16, 102–108 (2005). 15598946 10.1093/annonc/mdi006

[b18] Bhargava R. I., Gerald W. L., Li A. R., Pan Q., Lal P. *et al.* EGFR gene amplification in breast cancer: correlation with epidermal growth factor receptor mRNA and protein expression and HER-2 status and absence of EGFR-activating mutations. Mod Pathol. 18, 1027–1033 (2005). 15920544 10.1038/modpathol.3800438

[b19] Hanawa M., Suzuki S., Dobashi Y., Yamane T., Kono K. *et al.* EGFR protein overexpression and gene amplification in squamous cell carcinomas of the esophagus. Int J Cancer. 118, 1173–1180 (2006). 16161046 10.1002/ijc.21454

[b20] Reuter C. W., Morgan M. A. & Eckardt A. Targeting EGF-receptor-signalling in squamous cell carcinomas of the head and neck. Br J Cancer. 96, 408–416 (2007). 17224925 10.1038/sj.bjc.6603566 PMC2360023

[b21] Hirsch F. R., Varella-Garcia M., Bunn P. A., Di Maria M. V., Veve R. *et al.* Epidermal growth factor receptor in non-small-cell lung carcinomas: correlation between gene copy number and protein expression and impact on prognosis. J Clin Oncol. 21, 3798–3807 (2003). 12953099 10.1200/JCO.2003.11.069

[b22] Jimeno A., Tan A. C., Coffa J., Rajeshkumar N. V., Kulesza P. *et al.* Coordinated epidermal growth factor receptor pathway gene overexpression predicts epidermal growth factor receptor inhibitor sensitivity in pancreatic cancer. Cancer Res. 68, 2841–2849 (2008). 18413752 10.1158/0008-5472.CAN-07-5200

[b23] Van Cutsem E., Köhne C. H., Hitre E., Zaluski J., Chang Chien C. R. *et al.* Cetuximab and chemotherapy as initial treatment for metastatic colorectal cancer. N Engl J Med. 360, 1408–1417 (2009). 19339720 10.1056/NEJMoa0805019

[b24] Paez J. G., Jänne P. A., Lee J. C., Tracy S., Greulich H. *et al.* EGFR mutations in lung cancer: correlation with clinical response to gefitinib therapy. Science. 304, 1497–1500 (2004). 15118125 10.1126/science.1099314

[b25] Duan X., Li H., Qiu Z., Joshi B. P., Pant A. *et al.* MEMS-based multiphoton endomicroscope for repetitive imaging of mouse colon. Biomed Opt Express. 6, 3074–3083 (2015). 26309768 10.1364/BOE.6.003074 PMC4541532

[b26] Hinoi T., Akyol A., Theisen B. K., Ferguson D. O., Greenson J. K. *et al.* Mouse model of colonic adenoma-carcinoma progression based on somatic Apc inactivation. Cancer Res. 67, 9721–9730 (2007). 17942902 10.1158/0008-5472.CAN-07-2735

[b27] Choi Y. S., Yoon S., Kim K. L., Yoo J., Song P. *et al.* Computational design of binding proteins to EGFR domain II. PLoS One. 9, e92513 (2014). 24710267 10.1371/journal.pone.0092513 PMC3977815

[b28] Rowan A. J., Lamlum H., Ilyas M., Wheeler J., Straub J. *et al.* APC mutations in sporadic colorectal tumors: A mutational “hotspot” and interdependence of the “two hits”. Proc Natl Acad Sci. 97, 3352–3357 (2000). 10737795 10.1073/pnas.97.7.3352 PMC16243

[b29] Lee S., Xie J. & Chen X. Peptides and peptide hormones for molecular imaging and disease diagnosis. Chem Rev. 110, 3087–3111 (2010). 20225899 10.1021/cr900361p PMC2868934

[b30] Thurber G. M., Schmidt M. M. & Wittrup K. D. Antibody tumor penetration: transport opposed by systemic and antigen-mediated clearance. Adv Drug Deliv Rev. 60, 1421–1434 (2008). 18541331 10.1016/j.addr.2008.04.012 PMC2820307

[b31] Wu C. H., Kuo Y. H., Hong R. L. & Wu H. C. α-Enolase-binding peptide enhances drug delivery efficiency and therapeutic efficacy against colorectal cancer. Sci Transl Med. 7, 290ra91 (2015). 10.1126/scitranslmed.aaa9391 26041708

[b32] Hilderbrand S. A. & Weissleder R. Near-infrared fluorescence: application to *in vivo* molecular imaging. Curr Opin Chem Biol 14, 71–79 (2010). 19879798 10.1016/j.cbpa.2009.09.029

[b33] Wang T. D. In-Vivo Microscopy in Translational Multi-Modality Optical Imaging. (ed. Intes X., Azar F.) 19–32 (Artech House, 2008).

[b34] Slivka A., Gan I., Jamidar P., Costamagna G., Cesaro P. *et al.* Validation of the diagnostic accuracy of probe-based confocal laser endomicroscopy for the characterization of indeterminate biliary strictures: results of a prospective multicenter international study. Gastrointest Endosc. 81, 282–290 (2015). 25616752 10.1016/j.gie.2014.10.009

[b35] Kim P., Chung E., Yamashita H., Hung K. E., Mizoguchi A. *et al.* *In vivo* wide-area cellular imaging by side-view endomicroscopy. Nat Methods. 7, 303–305 (2010). 20228814 10.1038/nmeth.1440 PMC2849759

[b36] Kang D., Carruth R. W., Kim M., Schlachter S. C., Shishkov M. *et al.* Endoscopic probe optics for spectrally encoded confocal microscopy. Biomed Opt Express. 4, 1925–1936 (2013). 24156054 10.1364/BOE.4.001925 PMC3799656

[b37] Liu L., Wang E., Zhang X., Liang W., Li X. *et al.* MEMS-based 3D confocal scanning microendoscope using MEMS scanners for both lateral and axial scan, Sensors Actuators A Phys. 215, 89–95 (2014). 10.1016/j.sna.2013.09.035 PMC4083856 25013304

[b38] Piyawattanametha W., Ra H., Qiu Z., Friedland S., Liu J. T. C. *et al.* *In vivo* near-infrared dual-axis confocal microendoscopy in the human lower gastrointestinal tract. J. Biomed. Opt. 17, 21102 (2012). 10.1117/1.JBO.17.2.021102 PMC3380818 22463020

[b39] Yin C., Glaser A. K., Leigh S. Y., Chen Y., Wei L. *et al.* Miniature *in vivo* MEMS-based line-scanned dual-axis confocal microscope for point-of-care pathology. Biomed. Opt. Express. 7, 251 (2016). 26977337 10.1364/BOE.7.000251 PMC4771446

[b40] Fields G. B. & Noble R. L. Solid phase peptide synthesis utilizing 9-fluorenylmethoxycarbonyl amino acids. Int J Pept Protein Res. 35, 161–214 (1990). 2191922 10.1111/j.1399-3011.1990.tb00939.x

