# Supplementary material for: Visualizing epithelial expression of EGFR in vivo with distal scanning side-viewing confocal endomicroscope
Source: Sci Rep. 2016 Nov 22;6:37315. doi: 10.1038/srep37315 (PMC5118792; doi:10.1038/srep37315)
Supplement: Supplementary Information [file srep37315-s1.pdf]

Visualizing epithelial expression of EGFR in vivo with distal scanning side-viewing confocal endomicroscope

Xiyu Duan,[1] Haijun Li,[2] Juan Zhou,[2] Quan Zhou,[1] Kenn R. Oldham,[3] and Thomas D. Wang[1,2,3]

[1]Department of Biomedical Engineering, University of Michigan, Ann Arbor, MI, USA, 48109

[2]Department of Internal Medicine, University of Michigan, Ann Arbor, MI USA, 48109

[3]Department of Mechanical Engineering, University of Michigan, Ann Arbor, MI, USA, 48109

Supplementary Figures

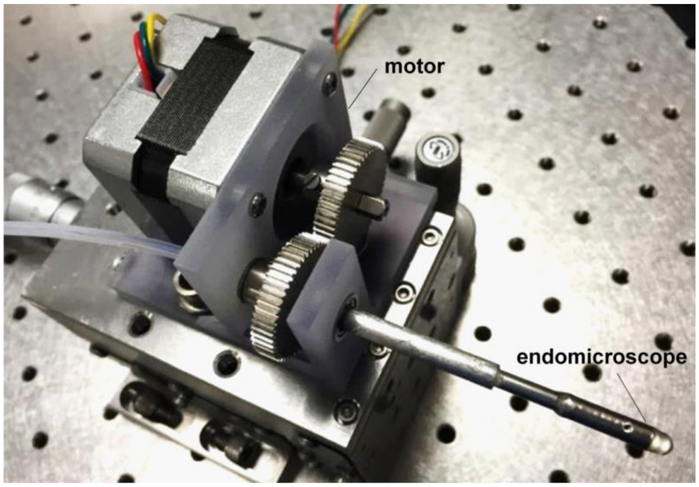

Fig. S1 – Motorized stage. We used a stepper motor to rotate and translate a platform to accurately position the distal tip of the side-viewing endomicroscope onto adenomas found with wide-field endoscopy using defined landmarks.

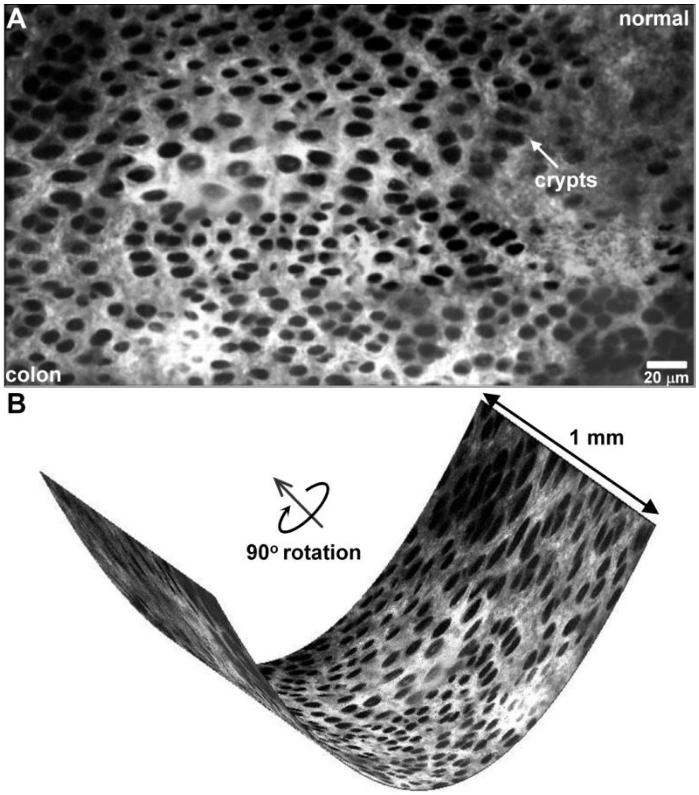

Fig. S2 – Panorama image. **A**) We used the side-viewing endomicroscope to greatly expand the image FOV from normal mouse colon in vivo by translating a distance of 1 mm and rotating by 90° at ~1 hour after systemic injection of Cy5.5. **B**) Mosaic image is warped using custom

469 software to mimic anatomy of colon wall.

Supplementary Video Legend

Visualization – Video shows real time images collected at 5 frames per sec of NIR fluorescence depicting EGFR expression from adenoma and adjacent normal colonic mucosa.
